# Mitigating Mucoadhesion of β–Cyclodextrins via PEGylation: Insights from ^19^F Diffusion NMR Analysis

**DOI:** 10.3390/ijms262311690

**Published:** 2025-12-02

**Authors:** Kim Trang Huu Nguyen, Yong Ba

**Affiliations:** Department of Chemistry and Biochemistry, California State University, Los Angeles, 5151 State University Drive, Los Angeles, CA 91016, USA; nqkimtranq@yahoo.com

**Keywords:** β-cyclodextrin, PEGylation, inclusion complexes, ^19^F diffusion NMR, ROESY NMR, kinetic diffusion model, mucin, mucoadhesion

## Abstract

β–Cyclodextrin (β–CD)-based materials are widely used in drug delivery, yet their interactions with mucosal barriers remain insufficiently understood. Because the mucus layer coating epithelial surfaces can hinder drug transport, elucidating β–CD–mucin interactions is critical for optimizing cyclodextrin-based carriers. In this study, we examined whether PEGylation can attenuate the mucoadhesive behavior of β–CD. Monomethoxy poly(ethylene glycol)-modified β–CDs (MPEG–β–CDs) were evaluated using ^19^F self-diffusion NMR spectroscopy coupled with a kinetic diffusion model describing reversible binding to stationary substrates. Mucin hydrogels were prepared from bovine submaxillary mucin and served as a model mucus environment. Diffusion coefficients were extracted from the ^19^F NMR signals of 1-fluoroadamantane (1FA) molecules encapsulated within HP-β–CD or MPEG–β–CD cavities. The results demonstrate that PEGylation substantially reduces β–CD–mucin adhesion, with longer PEG chains (2000 Da) providing more effective steric shielding than shorter chains (500 Da). These findings indicate that PEGylation can protect β–CD-included drugs during transport across mucosal barriers by minimizing unwanted β–CD–mucin interactions.

## 1. Introduction

Cyclodextrins (CDs) are cyclic oligosaccharides composed of α-(1,4)-linked glucose units that act as efficient solubilizing agents by forming non-covalent inclusion complexes with hydrophobic drugs [1,2]. They are classified by ring size: α-cyclodextrin (α–CD) contains six glucose units, β-cyclodextrin (β–CD) seven, and γ-cyclodextrin (γ–CD) eight, resulting in progressively larger internal cavities. CDs exhibit a truncated cone-shaped structure with a hydrophobic cavity and hydrophilic exterior due to peripheral hydroxyl groups [3]. This amphiphilic nature enables CDs and their derivatives to encapsulate poorly soluble drugs, making them widely used in pharmaceutical formulations [1,4,5,6,7,8,9,10,11,12]. Among them, β–CD is the most extensively applied. β-Cyclodextrin and many of its derivatives (e.g., hydroxypropyl-β-CD, methylated-β-CD) are widely used as pharmaceutical excipients due to their favorable biocompatibility and low toxicity [13]. β–CD inclusion can markedly enhance drug solubility and stability, facilitating oral delivery of otherwise insoluble compounds [9,14]. This inclusion complexation improves the drug’s solubility in aqueous environments, protects it from metabolic degradation, and enhances its bioavailability [15,16,17,18,19,20,21,22,23]. In recent years, β–CD and its derivatives (collectively denoted as β–CDs) have demonstrated great potential in various biomedical applications, including targeted drug delivery, controlled release systems, and reduced drug toxicity [1,24,25,26,27]. However, native β–CD has limited water solubility and potential nephrotoxicity, restricting clinical use [3,14]. To address these issues, chemical modifications have been developed to improve its solubility and inclusion efficiency. Here, we focus on PEGylation with monomethoxy poly(ethylene glycol) (MPEG), aiming to improve solubility, biocompatibility, and biodegradability [28,29,30,31]. Our previous work [32] demonstrated that MPEG–β–CD conjugates, synthesized via stable ether (C–O–C) linkages, exhibited significantly enhanced water solubility while maintaining β–CD’s inclusion capability.

PEGylation is a widely used strategy to modulate the physicochemical and biological properties of diverse substrates [33]. Notably, PEG coatings, especially with low-molecular-weight PEGs, have been shown to reduce mucoadhesion of nanoparticles and enhance their penetration through human mucus [34,35,36,37,38,39,40]. In addition, PEGylated drugs often display greater stability across varying pH and temperature conditions compared to their unmodified counterparts [41]. Thus, we expected that PEGylated β–CDs would protect the included drugs during transport through mucus.

Mucus is a complex, viscous, and adhesive secretion synthesized by specialized goblet cells within the columnar epithelium. It forms a continuous protective layer covering the respiratory, gastrointestinal, reproductive, and oculo-rhino-otolaryngeal tracts. Mucus is a heterogeneous mixture of several secretions and exhibits distinctive rheological properties, including viscoelasticity and shear-dependent viscosity. This dynamic barrier protects the underlying epithelium from a wide range of aggressors, such as pathogens and chemical or mechanical irritants, while simultaneously permitting selective exchange between luminal contents and the epithelial surface. As the first interface encountered by nutrients and orally administered therapeutics, mucus plays a critical role in modulating diffusion and absorption processes prior to systemic circulation. Moreover, it entraps microorganisms, thereby reducing their capacity to colonize the epithelial surface. Mucus is composed of approximately 95% water and contains salts, lipids (including fatty acids, phospholipids, and cholesterol), and proteins [42]. The primary macromolecular component responsible for its gel-like viscoelastic properties is the high-molecular-weight glycoprotein mucin, which forms the structural and functional backbone of the mucus network [43]. Therefore, understanding the diffusion properties of β–CDs and MPEG-β–CDs in mucus is critical for evaluating their potential as drug delivery vehicles.

Nuclear magnetic resonance (NMR) spectroscopy provides powerful, noninvasive approaches for probing molecular structure and dynamics in solution. In particular, diffusion NMR enables direct measurement of molecular mobility by monitoring translational diffusion coefficients, allowing quantitative assessment of complex formation, aggregation, and molecular transport [44,45,46,47,48]. Numerous studies have used NMR spectroscopy to quantify host-guest structure and transport [49]. For example, diffusion NMR and DOSY NMR experiments have enabled estimation of complex size and binding-dependent mobility, while ROESY/NOESY cross-peaks between guest protons and the cavity protons (H3/H5) of β-cyclodextrin provide direct through-space evidence of inclusion geometry [50,51,52,53]. A recent study using NMR and other methods illustrated cyclodextrin-mediated solubilization and binding analysis for baricitinib, highlighting how native CDs modulate drug behavior in aqueous media [54]. Complementary work has used ROESY-based mapping of adamantane–cyclodextrin contacts and diffusion-based analyses of small-molecule transport in complex media [55,56,57]. These precedents collectively motivate our use of ^19^F diffusion NMR and ROESY to quantify inclusion and transport in mucin hydrogels [44].

The objective of this study is to assess whether MPEG–β-cyclodextrins (MPEG–β–CDs) exhibit reduced mucoadhesive properties compared to β–CD derivatives including 2HP-β–CDs [58,59]. Our results demonstrate that PEGylation lowered adhesion to mucinous hydrogels, as evidenced by ^19^F diffusion NMR data analyzed using a kinetic model that describes molecular diffusion coupled with reversible binding to stationary substrates. The ^19^F NMR signals originated from 1-fluoroadamantane molecules encapsulated within β–CD cavities. These findings indicate that PEGylation effectively reduces mucoadhesion, thereby shielding β–CD-encapsulated drugs from interactions with mucus barriers that could otherwise lower drug concentration at the epithelial surface, increase metabolic degradation of drugs, and ultimately impair absorption.

## 2. Results

The ^1^H NMR spectra of 1FA in CDCl_3_, 2HP-β–CD in D_2_O, and MPEG_550_-β–CD in D_2_O are provided in Figures S1–S3 of the Supplementary Materials. Proton resonances were assigned according to the structural labels indicated in each spectrum. The spectrum of MPEG_2000_-β–CD is not included, as it is highly similar to that of MPEG_550_-β–CD, differing primarily in the increased intensity of the PEG-derived signals resulting from its longer polymer chain.

**Figure 1:**
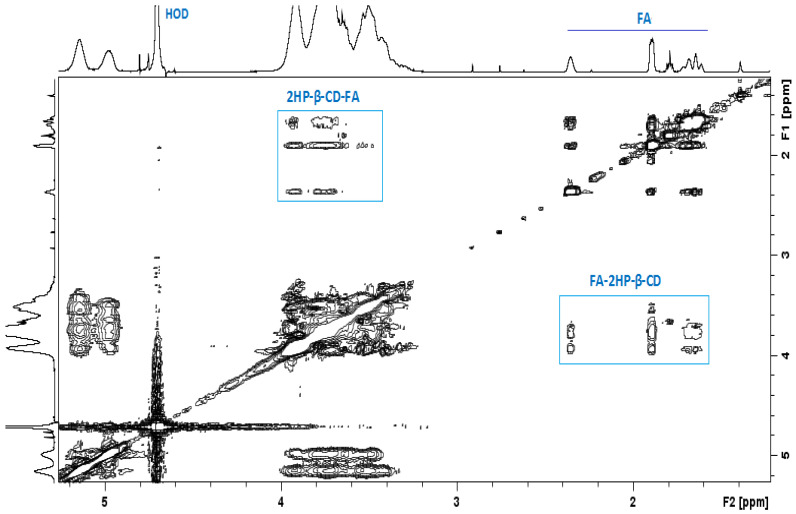
^1^H ROESY NMR spectrum of 1FA-2HP-β–CD IC dissolved in D_2_O solvent.

**Figure 2:**
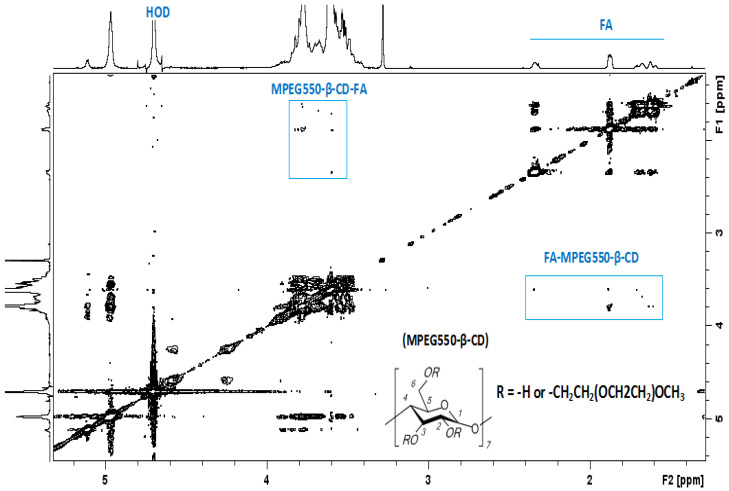
^1^H ROESY NMR spectrum of 1FA-MPEG_550_-β–CD IC dissolved in D_2_O solvent.

The 2D ^1^H ROESY spectra of the 1FA–2HP-β–CD and 1FA–MPEG_550_-β–CD inclusion complexes are shown in Figure 1 and Figure 2, respectively. The horizontal and vertical axes display the corresponding 1D ^1^H NMR spectra, while the 2D contour plots reveal cross-peaks arising from through-space dipolar interactions. Distinct cross-peaks (highlighted in blue boxes) were observed between the adamantane protons of 1FA and the H3 and H5 protons located inside the β–CD cavity. These correlations, reflecting proton–proton proximities of <~4 Å, confirm that 1FA is encapsulated within the β–CD cavity in aqueous solution. Moreover, the similar cross-peak patterns and relative intensities for the 1FA–2HP-β–CD and 1FA–MPEG_550_-β–CD complexes indicate comparable inclusion geometries and penetration depths, demonstrating that PEGylation does not significantly alter the binding mode within the β–CD cavity.

It is important to consider solvent effects when interpreting the chemical shifts of 1FA. In CDCl_3_, a nonpolar solvent, the adamantane protons of 1FA appear relatively shielded, producing upfield resonances. Upon encapsulation in β–CD cavities and measurement in D_2_O, a polar protic solvent, the environment of 1FA changes significantly. The hydrophobic cavity of β–CD partially shields 1FA from the bulk aqueous phase, but polarity differences still influence the chemical shifts. The observed downfield perturbations of the 1FA resonances in β–CD and D_2_O environment relative to CDCl_3_ thus reflect both solvent polarity and host–guest interactions. These effects are consistent with inclusion inside the cavity, where 1FA is less exposed to the polar solvent but still subject to deshielding from cavity protons and local anisotropy. Shifts were also detected for the H3 and H5 protons of β–CD, indicating their close spatial proximity to the guest molecule. Together, these chemical shift changes and ROESY cross-peaks provide complementary evidence for stable inclusion complexation in aqueous media.

Comparison of the 1FA–2HP-β–CD and 1FA–MPEG_550_-β–CD inclusion complexes shows that PEGylation does not interfere with the host–guest interactions. Both systems exhibit nearly identical ^1^H chemical-shift perturbations of 1FA (Δδ ≈ 0.10–0.12 ppm) upon complexation and display comparable ROESY cross-peak intensities between the adamantane protons of 1FA and the H3/H5 cavity protons of β–CD. These quantitative NMR features indicate that 1FA occupies a similar position within the hydrophobic cavity in both complexes and that the binding geometry and strength are preserved following PEG modification. Similar inclusion behavior was observed for MPEG_2000_-β–CD, further confirming that PEGylation primarily affects solubility and mucoadhesive properties rather than the intrinsic inclusion capability of β–CD.

Overall, these findings demonstrate that MPEG-β–CDs efficiently encapsulate hydrophobic guests such as 1FA while enhancing aqueous solubility. PEGylation improves the physicochemical properties of β–CD without disrupting its ability to form stable inclusion complexes, making MPEG-β–CD conjugates promising carriers for drug delivery through mucus and other biological barriers.

The ^19^F diffusion NMR experiment was performed using the Pulsed Field Gradient Stimulated Echo (PFGSE) technique [48]. This method produces a series of NMR spectra in which the signal intensity decreases with increasing pulsed field gradient (PFG) strength, according to Equation (1):

*I = I*_0_*Exp(−Dγ*^2^*g*^2^*δ*^2^(∆ − *δ*/3)) (1)

where *I* and *I*_0_ represent the echo intensities with and without the gradient, respectively; *D* is the diffusion coefficient (m^2^/s); *γ* is the gyromagnetic ratio of the observed nucleus (for ^19^F, *γ* ≈ 2.516 × 10^8^ rad·s^−1^·T^−1^); *δ* is the gradient pulse duration (s); Δ is the diffusion time (s); and *g* is the PFG strength (T/m (tesla per meter)).

**Figure 3:**
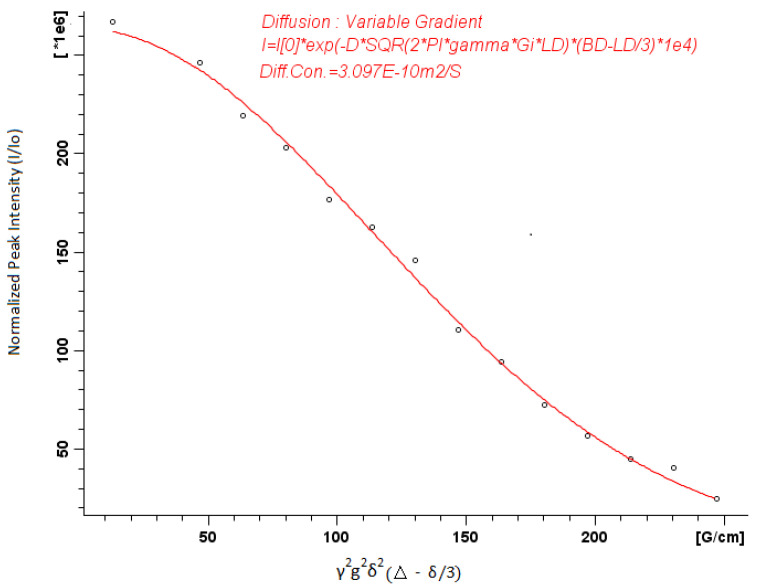
^19^F diffusion NMR result of 1FA–MPEG_550_–β–CD in a 5.0 mg/mL mucinous hydrogel. The circles represent the experimental data, and the solid line shows the theoretical fit to Equation (1). The diffusion coefficient obtained from the Stejskal–Tanner analysis is 3.10 × 10^−10^ m^2^/s. In the Bruker TopSpin display, two numerical factors appear during fitting. First, the notation 1e6 (i.e., 1 × 10^6^) is applied to the horizontal axis as a simple graphical scaling of the diffusion parameter *b = γ^2^g^2^δ^2^(*Δ *− δ/3)*, allowing large *b*-values to be presented in a compact numeric range. Second, the 1e4 (i.e., 1 × 10^4^) factor within the internal equation arises from the conversion of gradient amplitudes from G/cm (instrument units) to T/m, as required by the Stejskal–Tanner equation, since 1 G/cm = 10^−4^ T/m. These software-based scaling and unit-conversion factors influence only the displayed labels and do not affect the calculated diffusion coefficient.

A representative diffusion decay curve for the 1FA–MPEG_550_–β–CD inclusion complex in a 5.0 mg/mL mucin hydrogel is shown in Figure 3. The circular points represent the experimental data, while the solid line depicts the theoretical fit obtained using Equation (1). The calculated diffusion coefficient was (3.10 ± 0.02) × 10^−10^ m^2^/s.

**Figure 4:**
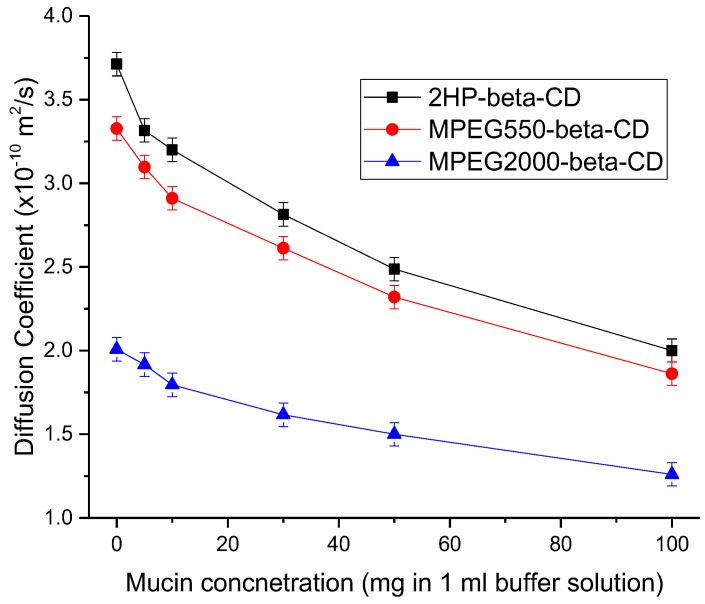
Diffusion coefficients of 1FA–2HP–β–CD, 1FA–MPEG_550_–β–CD, and 1FA–MPEG_2000_–β–CD as a function of mucin concentration in mucinous hydrogels.

Figure 4 presents the diffusion coefficients of 1FA–2HP–β–CD, 1FA–MPEG_550_–β–CD, and 1FA–MPEG_2000_–β–CD inclusion complexes in mucinous hydrogels at different mucin concentrations. For all ICs, the diffusion coefficients decreased with increasing mucin concentration, indicating that higher mucin levels hinder molecular diffusion. At the same mucin concentration, the diffusion coefficients decreased in the order: 1FA–2HP–β–CD > 1FA–MPEG_550_–β–CD > 1FA–MPEG_2000_–β–CD.

## 3. Discussion

Diffusion coefficients of the inclusion complexes (ICs) in mucus were governed by three principal factors: (i) the molecular size of the ICs, (ii) the physical hindrance imposed by the mucin polymer network, and (iii) reversible binding interactions between the ICs and mucin binding sites. To distinguish the effects of physical hindrance and mucin interactions from those attributable purely to molecular size, a normalized diffusion coefficient (*D*_normalized_) was used. This parameter is defined as the ratio of the diffusion coefficient measured in mucus (*D*_mucus_) to that measured in buffer (*D*_buffer_) under identical conditions:

(2) $$D_{normalized}=\frac{D_{mucus}}{D_{buffer}}$$

Normalizing *D*_mucus_ by the corresponding *D*_buffer_ for the same inclusion complex removes intrinsic contributions from hydrodynamic size, molecular shape, and solvent viscosity, all of which affect both measurements equally. As a result, the normalized value emphasizes mucin-specific effects—such as transient binding, partitioning, and mesh obstruction—that selectively slow diffusion in the mucus environment. Similar normalization strategies are well-established in diffusion NMR and biophysical transport studies and have been previously applied to cyclodextrin-based systems in mucus and other complex biological media [59,60,61,62].

**Figure 5:**
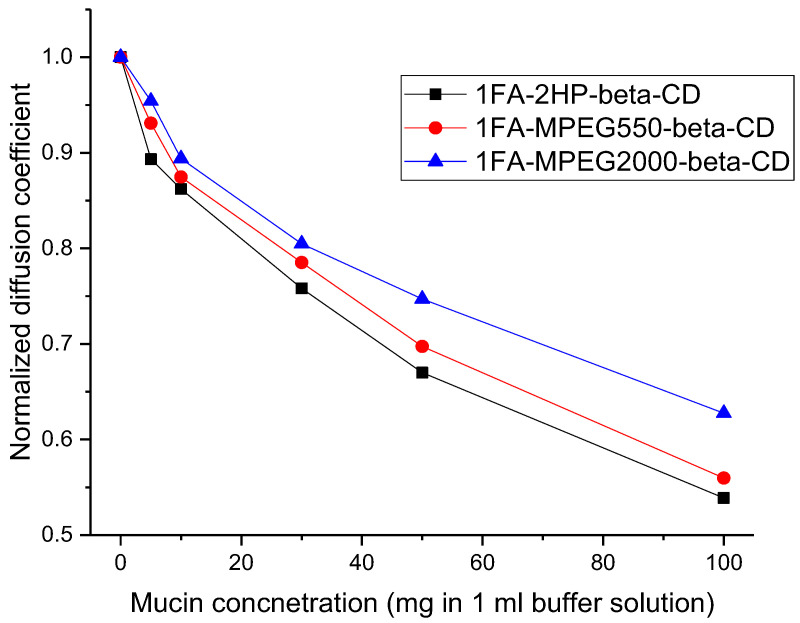
Normalized diffusion coefficients of 1FA–2HP–β–CD, 1FA–MPEG_550_–β–CD, and 1FA–MPEG_2000_–β–CD inclusion complexes as a function of mucin concentration.

The normalized diffusion coefficients are presented in Figure 5. The 1FA–2HP–β–CD ICs exhibited lower values than the 1FA–MPEG–β–CDs at equivalent mucin concentrations, indicating that 2HP–β–CD binds more strongly to the mucin network. In contrast, both MPEG–β–CD ICs showed reduced mucoadhesive interactions, consistent with the shielding effect of PEG chains on the β–CD surface. Furthermore, the 1FA–MPEG_2000_–β–CD ICs displayed higher *D_normalized_* values than 1FA–MPEG_550_–β–CD, suggesting that the longer PEG chains more effectively diminish mucoadhesive interactions. We infer that the binding effect, rather than simple physical hindrance by the mucin network, primarily governs the observed diffusion behavior, since a purely steric effect would predict lower normalized diffusion coefficients for the larger MPEG_2000_–β–CDs. These findings support the conclusion that PEGylation, particularly with the higher-molecular-weight MPEG (2000 Da), significantly reduces β–CD–mucin adhesion, thereby enabling safer and more efficient transport of β–CD-included drugs through mucus-like environments.

In the following discussion, we assume the bindings of ICs to the mucin networks are dominant and the physical hindrance effect is negligible. To further evaluate whether steric obstruction contributes significantly to the decrease in diffusion, we refer to our previous analysis of mucin mesh sizes [59] using Ogston’s and Renkin’s models. The average mesh diameters of bovine submaxillary mucin range from approximately 600 nm to 370 nm at 10–100 mg/mL mucin concentrations, respectively. In addition, a study using electron microscopy on cervical mucus confirmed that the mesh spacing between mucin fibers is 20 nm to 200 nm [63]. These dimensions are two orders of magnitude larger than the hydrodynamic size of 1FA–β–CD inclusion complexes (~1.5–2.0 nm), meaning that steric hindrance alone would account for only minimal diffusional impedance. In contrast, the experimentally observed decrease in normalized diffusion coefficients (*D_normalized_*: MPEG2000 > MPEG550 > HP-β–CD) and the size-trend inversion strongly support a diffusion model dominated by reversible IC–mucin binding rather than physical sieving.

The root-mean-square displacement (*r*_rms_) of a particle diffusing in three-dimensional space is given by:

(3) $$r_{rms}=\;\sqrt{6Dt}$$

where *D* is the diffusion coefficient and *t* is the diffusion time. To maintain the same *r_rms_* for an IC in buffer and in mucus (the mucinous hydrogel), the diffusion time in mucus (*t_mucus_*) must be longer than that in buffer (*t_buffer_*), as expressed by

(4) $$r_{rms,\;buffer}=\sqrt{6D_{buffer}t_{buffer}}{\;=r}_{rms,\;mucus}=\sqrt{6D_{mucus}t_{mucus}}$$

which gives

(5) $$\frac{t_{buffer}}{t_{mucus}}=\frac{D_{mucus}}{D_{buffer}}$$

Thus, the average fractional dwell time of an IC residing at mucin binding sites is defined as

(6) $$f_{mucus}=\frac{{t_{mucus}-t}_{buffer}}{t_{mucus}}=\frac{{D_{buffer}-D}_{mucus}}{D_{buffer}}$$

Similarly, the fractional dwell time of the IC diffusing freely in the buffer is given by

(7) $$f_{buffer}=1-f_{mucus}=\frac{t_{buffer}}{t_{mucus}}=\frac{D_{mucus}}{D_{buffer}}$$

The fractional dwell time, *f_mucus_*, also represents the fraction of IC molecules bound to mucin binding sites within the hydrogel at equilibrium because diffusion NMR measures molecular self-diffusion under thermodynamic equilibrium.

**Figure 6:**
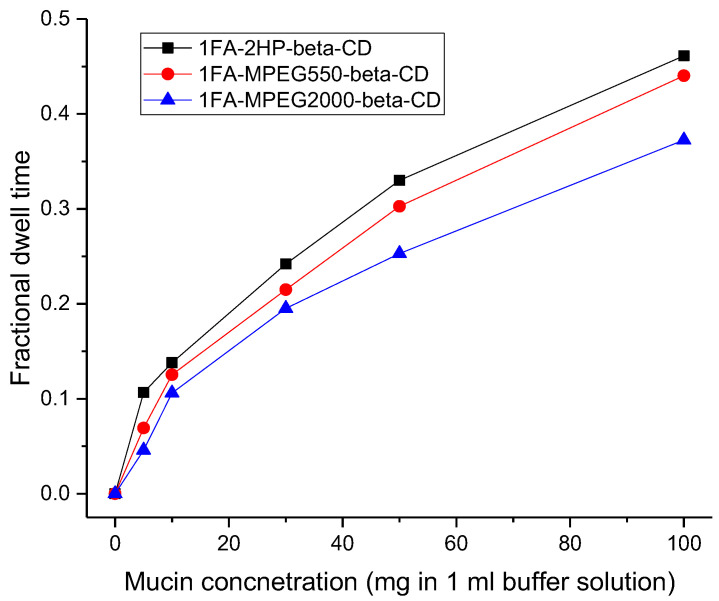
Fractional dwell times of 1FA-2HP-β–CD, 1FA-MPEG_500_-β–CD and 1FA-MPEG_2000_-β–CD versus mucin concentrations in the mucinous hydrogel.

Figure 6 shows the fractional dwell times, *f_mucus_*, of 1FA–2HP–β–CD, 1FA–MPEG_500_–β–CD, and 1FA–MPEG_2000_–β–CD as a function of mucin concentration. All inclusion complexes (ICs) exhibited increasing fractional dwell times with increasing mucin concentration, indicating a larger population of IC molecules bound to mucin binding sites as the number of available sites increased. The observed order of fractional dwell times was 1FA–2HP–β–CD > 1FA–MPEG_500_–β–CD > 1FA–MPEG_2000_–β–CD at any given mucin concentration. Larger fractional dwell times among the ICs correspond to stronger binding affinities between the ICs and mucin. These results demonstrate that both the molecular structure of the ICs and the mucin concentration significantly influence the fraction of mucin-bound ICs within the hydrogel matrix.

We model the reversible binding of an inclusion complex *A* to mucin sites *M* as:

*A + M* ⇌ *AM* (8)

which obeys mass-action kinetics with association rate *v_+_ = k_+_[A][M]* (bimolecular) and dissociation rate *v_−_ = k_−_[AM]* (unimolecular). At equilibrium, the net rate is zero, so *v_+_ = v_−_* and

*k_+_[A][M] = k_−_[AM]* (9)

where *k_+_* and *k_−_* denote the forward and reverse rate constants, respectively. Using conservation *[A*_0_*] = [A] + [AM] and [M*_0_*] = [M] + [AM]*, this becomes

*k_+_([A*_0_*] − [AM])([M*_0_*] − [AM]) = k_−_[AM]* (10)

where *[A*_0_*]* and *[M*_0_*]* denote the initial concentration of *A*, and the initial concentration of mucin binding sites, respectively. The equilibrium association constant (*K_a_*) and dissociation constant (*K_d_*) are then

(11) $$K_{a}=\frac{1}{K_{d}}=\frac{\left[AM\right]}{\left[A\right][M\}}=\frac{k_{+}}{k_{-}}$$

For diffusion coupled with binding to stationary sites, the equation of mass conservation can be written as:

(12) $$D_{buffer}\nabla^{2}\left[A\right]-\frac{\partial \left[AM\right]}{\partial t}=\;\frac{\partial \left[A\right]}{\partial t}$$

where *D_buffer_* denotes the diffusion coefficient of *A* in buffer. We further define the binding constant as:

(13) $$K_{b}=\frac{\left[AM\right]}{\left[A\right]}$$

Substituting [*AM*] in Equation (13) into Equation (12) under equilibrium conditions yields:

(14) $$\frac{D_{buffer}}{K_{b}+1}\nabla^{2}\left[A\right]=\;\frac{\partial \left[A\right]}{\partial t}$$

As a result, the apparent diffusion coefficient of the IC, *D_mucus_*, measured in the mucinous hydrogels is given by:

(15) $$D_{mucus}=\frac{D_{buffer}}{K_{b}+1}=\frac{D_{buffer}}{K_{a}[M]+1}=\frac{D_{buffer}}{[M]/K_{d}+1}$$

This leads to the relationship:

(16) $$K_{b}=\frac{D_{buffer}-D_{mucus}}{D_{mucus}}=\frac{f_{mucus}}{f_{buffer}}=\frac{f_{mucus}}{1-f_{mucus}}$$

Comparison of Equations (13) and (16) indicates that the ratio of the fractional dwell times of an IC in mucus and in buffer equals *K_b_*, i.e., the ratio of the concentration of bound ICs to the concentration of free ICs in buffer for an ensemble of IC molecules in the hydrogel system at chemical equilibrium.

**Figure 7:**
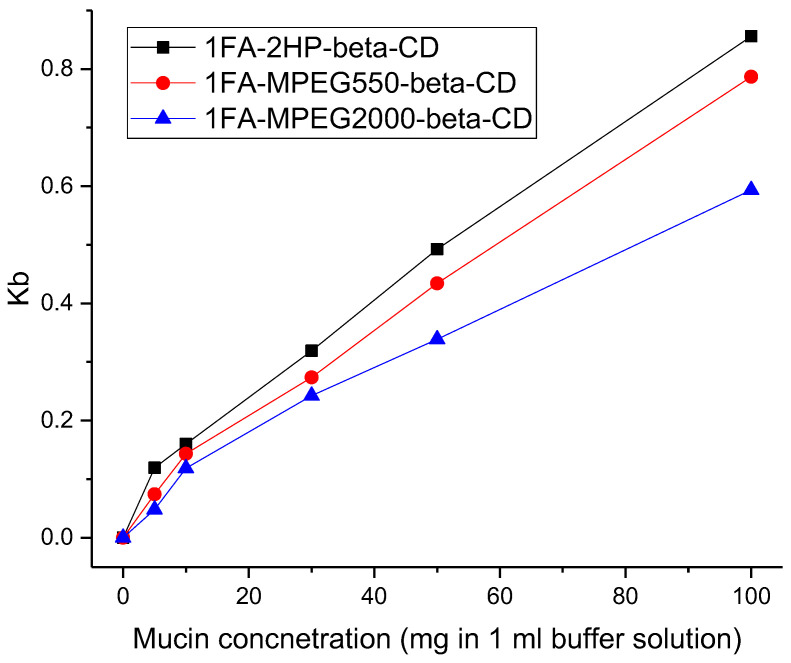
Binding constants (*K_b_*) for the inclusion complexes (ICs) interacting with mucin binding sites in mucinous hydrogels at different concentrations.

Figure 7 presents the *K_b_* values of the ICs in mucinous hydrogels at various mucin concentrations. All *K_b_* values increase with increasing mucin concentration, reflecting the greater availability of binding sites. At any given mucin concentration, the observed order of binding constants is: 1FA–2HP–β–CD > 1FA–MPEG_500_–β–CD > 1FA–MPEG_2000_–β–CD. A higher *K_b_* indicates a stronger binding affinity of the IC toward mucin under the same mucin conditions. In this study, the binding sites are hypothetical, and their exact number is unknown, although the binding constants were extracted from the ^19^F diffusion NMR data. By combining Equations (11) and (13), the following relationship is obtained:

*K_b_ = K_a_[M] = K_a_([M*_0_*] − [AM])* (17)

When the initial mucin concentration, *[M_0_]*, is much larger than the β–CD concentration, (*[M*_0_*] − [AM]*) approximates *[M*_0_*].* Thus, *K_b_* is expected to increase linearly with mucin concentration. The trends observed in Figure 7—particularly at higher mucin concentrations—support this prediction and validate the applicability of our diffusion-based binding model. Therefore, the kinetics of binding of ICs to mucin can be approximated as pseudo-first-order under our experimental conditions. Overall, the binding constant analysis indicates that the MPEG–β–CD derivatives exhibit weaker adhesive interactions with mucin compared to 2HP–β–CD.

Figure 7 indicates that the binding between the inclusion complexes (ICs) and mucin is strong. However, this does not imply that mucin and the ICs should exhibit similar diffusion coefficients. In mucus hydrogels, mucins form a highly entangled, partially cross-linked polymer network with extremely slow self-diffusion. Reported mucin diffusivities are on the order of 10^−13^–10^−14^ m^2^/s [63,64,65], depending on concentration and biological source. These values are three to four orders of magnitude lower than the diffusion coefficients of the 1FA–β–CD inclusion complexes measured in this study (~10^−10^ m^2^/s). Consequently, mucin is effectively immobile on the diffusion NMR timescale (tens of milliseconds), serving as a stationary binding scaffold rather than a freely diffusing species. Within this framework, the observed decrease in the apparent IC diffusion coefficient arises from intermittent, reversible binding of ICs to immobilized mucin sites, which increases the fraction of time spent in the bound (slow) state. This mechanism is fully consistent with classical diffusion–binding models and does not require mucin and ICs to co-diffuse. As shown in the single-exponential diffusion decays (Figure 3), the PFG-NMR attenuation curves fit a mono-exponential Stejskal–Tanner model, indicating fast exchange between free and bound states relative to diffusion time. Therefore, direct measurement of mucin self-diffusion is not necessary for quantifying IC–mucin interactions in this system.

In a related diffusion NMR study [59], we found that 2HP–β–CD and 2-carboxyethyl–β–CD exhibited the weakest adhesive interactions with mucin among the five β–CD derivatives examined, which also included 2,3,6-tri-O-methyl–β–CD, 6-monodeoxy-6-monoamino–β–CD, and a β–CD polymer containing three to five β–CD units. The observed order of binding constants (*K_b_*) in this study is 1FA–2HP–β–CD > 1FA–MPEG_500_–β–CD > 1FA–MPEG_2000_–β–CD. Thus, MPEG_2000_–β–CDs demonstrated the lowest adhesive interactions among these β–CD derivatives. Other reports have shown that surface modification of nanoparticles with low-molecular-weight poly(ethylene glycol) (PEG, 2.0–3.4 kDa compared to PEG > 10 kDa) reduces mucoadhesion and enhances particle penetration through fresh, undiluted human mucus at rates only up to fourfold lower than in pure water [35,36]. In contrast, coating particles with higher–molecular–weight PEG (>10 kDa) renders them mucoadhesive [35,36], due to the ability of long PEG chains to interpenetrate the mucus network [36,37,38,39,40,66]. We believe that PEG_2000_ offers an optimal balance between synthetic accessibility for preparing PEGylated β–CDs and effective shielding of β–CDs in the mucus environment.

Published diffusion coefficients of β-cyclodextrins in mucus are scarce. To our knowledge, the most directly comparable work is our previous work [59], where we measured the diffusion of 1FA–β–CD inclusion complexes in bovine submaxillary and human nasal mucus using ^19^F diffusion NMR and a kinetic binding–diffusion model. We reported that 2HP–β–CD exhibited relatively weak mucin binding among five β–CD derivatives. Our diffusion-derived *D_normalized_* and *K_b_* trends for MPEG–β–CDs are consistent with this behavior. Other studies on functionalized cyclodextrins (e.g., thiolated β–CDs) generally report mucodiffusion, retention time, or permeation rather than absolute diffusion coefficients, and therefore cannot be directly compared numerically [67]. For a broader context, diffusion of hydrophobic molecules in native mucus has been shown to decrease by up to ~50–60% compared to buffer (e.g., testosterone in porcine intestinal mucus) [68], demonstrating the strong influence of mucin–solute interactions on molecular transport.

Mucin’s heterogeneous structure presents multiple interaction modes for β-cyclodextrins (β–CDs) and their derivatives, including hydrogen bonding via hydroxyl-rich domains, electrostatic contacts with charged glycans, and hydrophobic interactions with non-polar protein patches or lipid-associated regions. Mucins are high–molecular–weight glycoproteins (0.5–20 MDa) composed of densely O-glycosylated protein backbones, with carbohydrates accounting for up to 80% of their total molecular weight. Secreted mucins [69], which dominate the rheological properties of mucus, contain serine- and threonine-rich domains that serve as attachment sites for N-acetylgalactosamine and additional sugars such as N-acetylglucosamine, galactose, fucose, sialic acids, and sulfate. These features give rise to a wide range of possible molecular interactions including hydrogen bonding, hydrophobic contacts, and electrostatic interactions [70] while the overall negative charge of mucin also enables selective binding to positively charged species [71] and can even produce anti-adhesive behavior toward anionic molecules. Mucins may additionally interact with lipids through hydrophobic domains [72] and can form enhanced mucoadhesive interactions via polyelectrolytic forces, hydrogen bonding, or disulfide linkages, properties [70,73,74] that have motivated their use as high–molecular–weight additives in drug delivery formulations such as artificial tears [75].

Within this biochemical framework, native β–CD interacts with mucin primarily through hydrogen bonding, whereas chemical substitutions on β–CD, such as hydroxypropylation, methylation, or PEGylation, modulate polarity, hydration, steric accessibility, and overall adhesive potential. Less-shielded derivatives can engage multiple mucin interaction modes. PEG chains present a hydrophilic, near-neutral, and highly hydrated corona that sterically and enthalpically disfavors adhesive contacts with mucin’s hydrophobic patches and charged domains. Dense PEG grafting minimizes polymer–polymer bridging and hydrogen-bonding with mucins, thereby reducing mucoadhesion and facilitating higher effective diffusivity. This behavior is well-documented for PEG-modified carriers in mucus [36,37,38,39,40,66] and underpins the higher *D_normalized_*. As a result, PEG–β–CDs interact more weakly with mucin and preferentially partition into the aqueous phase, effectively reducing mucin adhesion compared to other β–CD derivatives.

Our results indicate that MPEG chains effectively shield β–CD from adhesive interaction with mucin, implying that drugs encapsulated in MPEG–β–CDs can be better protected during mucus penetration. Cyclodextrins are already widely used as drug carriers due to their ability to enhance aqueous solubility, improve drug stability, and enable controlled release [7,76,77]. About 130 cyclodextrin-based pharmaceutical products are currently on the global market [1,8,9,10]. We believe that our PEGylated β–CDs, with their improved physicochemical and muco-inert properties, hold promising potential for pharmaceutical and broader industrial applications.

## 4. Materials and Methods

2-Hydroxypropyl-β-cyclodextrin (2HP-β–CD, CAS#: 128446-35-5), monomethoxy poly(ethylene glycol) (MPEG, CAS#: 9004-74-4), and tetrahydrofuran (THF, CAS#: 109-99-9) were purchased from Sigma-Aldrich (Burlington, MA, USA). 1-Fluoroadamantane (1FA, CAS#: 768-92-3) was obtained from TCI America (Portland, OR, USA). Deuterium oxide (D_2_O, CAS#: 7789-20-0) and deuterium chloroform (CDCl_3_, CAS#: 865-49-6) were purchased from Cambridge Isotope Laboratories, Inc. (Tewksbury, MA, USA). Bovine submaxillary mucin (BMS, CAS#: 84195-52-8) was purchased from Sigma-Aldrich. Phosphate buffer (pH 7.0, 0.43 M, CAS#: 5808-16) was obtained from Ricca Chemical (Arlington, TX, USA).

The MPEG–β–CD conjugates were synthesized from monomethoxy poly(ethylene glycol) (MPEG) and β-cyclodextrin following our previously reported procedure [32]. Mechanistically, the reaction proceeds through a nucleophilic substitution (SN2) pathway. First, MPEG was activated by converting its terminal hydroxyl group into a better leaving group using p-toluenesulfonyl chloride in the presence of pyridine, yielding tosylated MPEG (MPEG–OTs). Separately, β–CD was dried and treated with sodium hydride in anhydrous N,N-dimethylformamide to selectively deprotonate the primary hydroxyl groups at the C-6 position, generating nucleophilic alkoxide species (β–CD–O^−^).

**Scheme 1:**
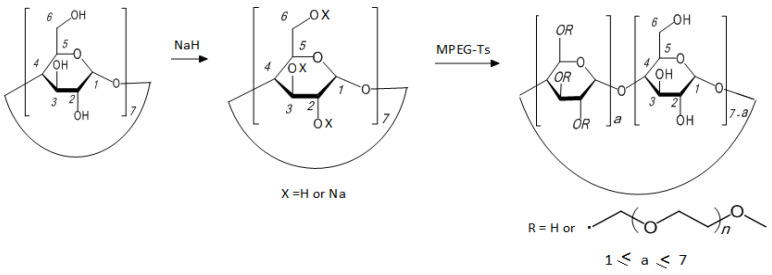
Synthesis pathway of PEG-modified β-cyclodextrin (PEG–β–CD). The number of glucose units conjugated to MPEG are labeled with “a” and conjugation may be randomly distributed among the seven glucose residues in the β-cyclodextrin ring.

As shown in Scheme 1, the activated MPEG–OTs was then added to the β–CD alkoxide solution, where the 6-O^−^ group of β–CD performed a nucleophilic attack on the electrophilic carbon adjacent to the tosylate group. This displaced the tosylate and afforded MPEG–β–CD linked through a stable ether (C–O–C) bond. The reaction mixture was stirred at 60–65 °C for several days, and the product was purified by precipitation in diethyl ether, dialysis against water, and lyophilization. Importantly, this conjugation strategy preserves the native toroidal cavity of β–CD, enabling the resulting MPEG–β–CD to retain its ability to encapsulate hydrophobic guest molecules.

The preparation of 1FA-2HP-β–CD and 1FA-MPEG-β–CD inclusion complexes (ICs) followed our previously reported method [32,58,59]. In short, 1FA was dissolved in THF, while MPEG-β–CDs or 2HP-β–CDs were dissolved in deionized water. The solutions were mixed using a vortex mixer and subsequently lyophilized to yield solid IC powders. The molar ratio of 1FA to MPEG-β–CD/2HP-β–CD was maintained at 1:1.

Mucin hydrogels (artificial mucus) were prepared from bovine submaxillary mucin dissolved in phosphate buffer (pH 7.0) containing sodium chloride (9.0 g/L) to mimic physiological saline concentration. Final mucin concentrations of 5.0, 10.0, 30.0, 50.0, and 100.0 mg/mL were prepared. For diffusion studies, 3.0 mM 1FA-MPEG-β–CD ICs were mixed with 3.0 mM MPEG-β–CDs in mucinous hydrogels. Similarly, 3.0 mM 1FA-2HP-β–CD ICs were prepared with 3.0 mM 2HP-β–CDs. Although adamantane derivatives have β–CD association constants (K_a_) on the order of 10^4^–10^5^ [78], additional free MPEG–β–CD and 2HP–β–CD were included to reinforce the encapsulation of 1FA within the β–CD cavities during diffusion in the mucinous hydrogels. Our previous results confirmed that β–CDs and their 1FA inclusion complexes exhibited identical diffusion coefficients when measured by both ^1^H and ^19^F NMR, demonstrating strong retention of 1FA within the cavities of β–CDs [58,59]. Therefore, the diffusion constants obtained from ^19^F diffusion NMR represent the translational motion of the entire ICs. Use of ^19^F signals, rather than ^1^H signals of β–CDs, was to avoid overlap with the abundant proton signals from water and mucin.

^1^H 1D and 2D ROESY NMR spectra were acquired on a Bruker BioSpin Avance™ II (Ettlingen, Germany) 400 MHz NMR spectrometer, and diffusion measurements were performed on a Bruker BioSpin Avance 600 MHz NMR spectrometer at room temperature. The HOD signal remaining in D_2_O at 4.800 ppm was used as the internal secondary reference for all ^1^H chemical shifts. For ^19^F NMR, hexafluorobenzene (C_6_F_6_) in benzene-d_6_ served as the external reference, with its resonance set to −164.9 ppm relative to CFCl_3_ at 0 ppm.

## Data Availability

The original contributions presented in this study are included in the article/Supplementary Material. Further inquiries can be directed to the corresponding author.

## Supplementary Materials

The following supporting information can be downloaded at: https://www.mdpi.com/article/10.3390/ijms262311690/s1.
