# Supplementary material for: Mitigating Mucoadhesion of β–Cyclodextrins via PEGylation: Insights from 19F Diffusion NMR Analysis
Source: Int J Mol Sci. 2025 Dec 2;26(23):11690. doi: 10.3390/ijms262311690 (PMC12692281; doi:10.3390/ijms262311690)
Supplement: Supplementary file 1 [file ijms-26-11690-s001.zip › ijms-3950698-supplementary.pdf]

Mitigating Mucoadhesion of β-Cyclodextrins via PEGylation: Insights from ^{19}F Diffusion NMR Analysis

Kim Trang Huu Nguyen and Yong Ba*

Department of Chemistry and Biochemistry, California State University, Los Angeles, 5151 State University Drive, Los Angeles, CA 91016, USA

The ^1H NMR spectra of 1FA in $CDCl_3$, 2HP-β-CD in D_2O, and $MPEG_{550}$-β-CD in D_2O are shown in Figures 1–3. Proton resonances were assigned according to the structural labels in the spectra. The spectrum of $MPEG_{2000}$-β-CD is not shown, as it closely resembles that of $MPEG_{550}$-β-CD, differing mainly in the larger PEG-derived peaks due to the longer polymer chain.

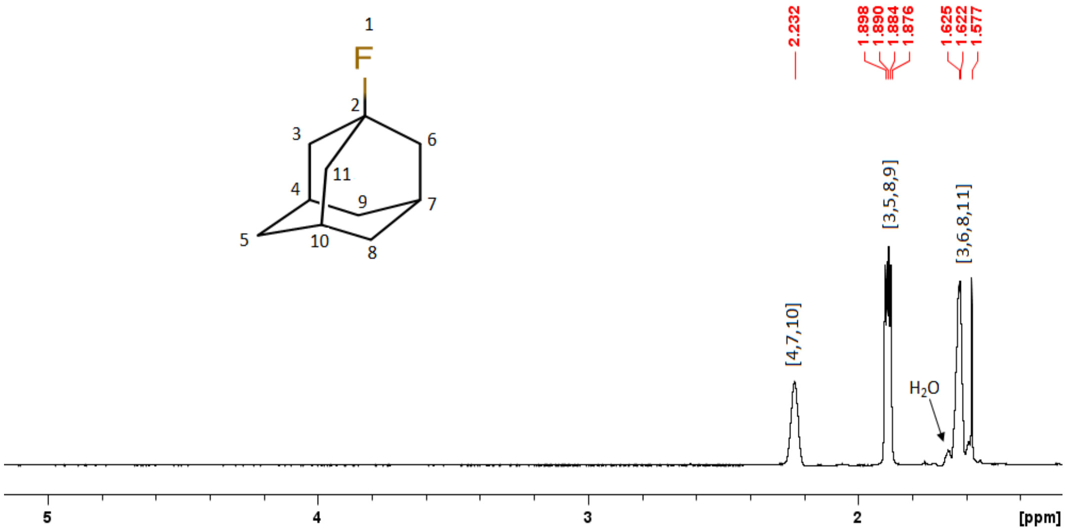

Figure S1. ^1H NMR spectrum of 1FA dissolved in $CDCl_3$ solvent.

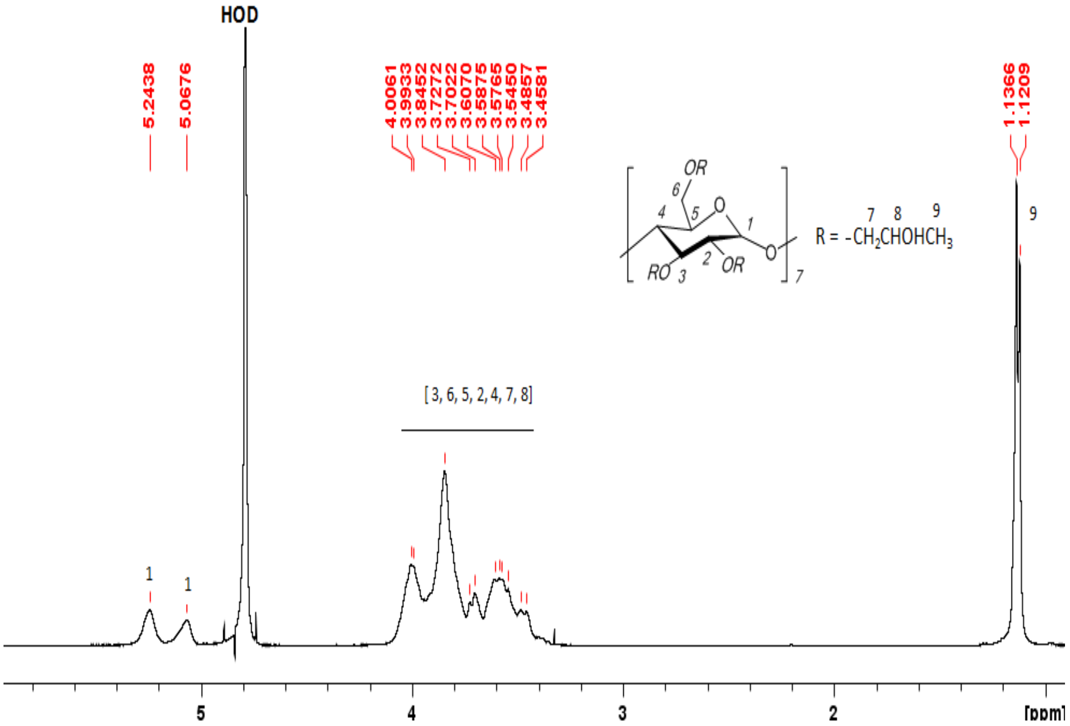

Figure S2. ^{1}H NMR spectrum of 2HP-β-CD dissolved in D_2O solvent.

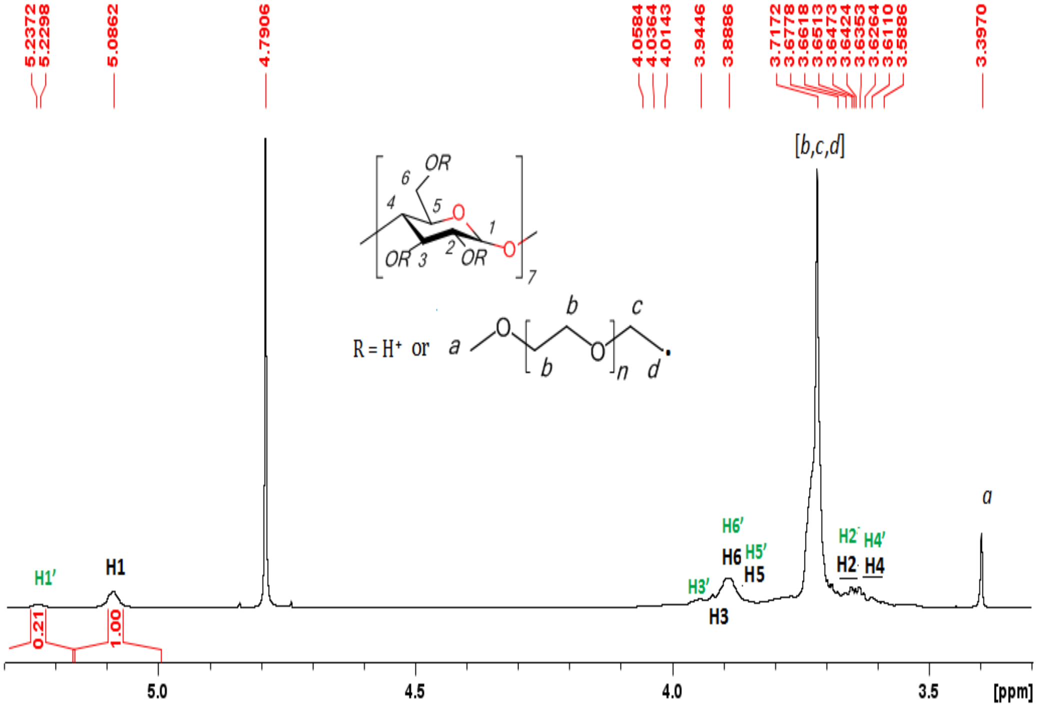

Figure S3. ^{1}H NMR spectrum of $MPEG_{550}$-β-CD dissolved in D_2O solvent.
